# Prescription drug use and potential teratogenicity risk among pregnant women attending maternal and child health clinic of Kemisse General Hospital, Northeast, Ethiopia

**DOI:** 10.1186/s13104-019-4641-1

**Published:** 2019-09-18

**Authors:** Belete Kassa Alemu, Nesredin Nigatu Wolle

**Affiliations:** 1 Department of Pharmacy, Pharmacology and Toxicology Unit, College of Medicine and Health Sciences, Wollo University, P.o.box 1145, Dessie, Ethiopia; 2 Kemisse General Hospital, Kemisse, Ethiopia

**Keywords:** Kemisse General Hospital, FDA drug risk category, Teratogenicity risk, Pregnant women

## Abstract

**Objective:**

To investigate medications prescribed for pregnant women and their potential teratogenicity risk in Kemisse General Hospital.

**Result:**

A total of 263 medical records of pregnant women were reviewed, of which 234 pregnant women were prescribed with a total of 430 prescription drugs. The average numbers of drugs per pregnant women was found to be 1.84. Most pregnant women 166 (63.2%) were in the third trimester and more than half of them (51.3%) were multigravida. The maximum number of drugs were prescribed in the second trimester 162 (37.67%) followed by third trimester 143 (33.26%). Supplemental drugs were the most widely used medications 297 (69.07%) and followed by 82 (19.1%) drugs from category B; 54 (12.6%) drugs from category C; and the rest 7 (1.6%) drugs from category D. There was no any drug from category X. Moreover, approximately one third of the pregnant women encountered with drugs from category B, C and D. However, there were no FDA category C and D drugs prescribed in first trimester.

## Introduction

Drugs use in pregnancy should always question two important queries and maintain a fine balance of them; no harm should be posed to the baby due to the drug, and no harm must come to the mother or baby because of inadequate treatment [1]. Medication use during pregnancy has been an issue of serious concern and needs monitoring since historical milestone of thalidomide disaster during the 1960s [2]. The physiologic, pharmacokinetic and pharmacodynamic changes occur during pregnancy requires special therapeutic consideration in pregnant and lactating women. Not only the changes have occurred, but also these physiologic changes are not fixed throughout pregnancy but rather reflect a continuum of change as pregnancy progresses. Moreover, pregnant women have been often excluded from clinical trials and extrapolation of pharmacokinetics data from studies performed in non-pregnant adults or evidences generated from animal studies are not often suitable [3]. Therefore, pregnancy management using medications has been challenging to both health care providers and pregnant women, given the fear of teratogenicity effects and the potential for fetal harms.

A large number of studies have reported that the utilization of a large number of drugs during pregnancy [4–8]. Substantial number of drugs was also used from Food and Drug administration (FDA) pregnancy risk category D and X [9]. Hence this study was carried out to evaluate potential teratogenicity risk and therefore improve prescription practices and avoids risks to fetus in women attending Maternal and Child Health (MCH) clinic of Kemisse General Hospital (KGH) in eastern Amhara, Ethiopia.

## Main texts

### Methods

#### Study area, design and period

The study was conducted at KGH, located in Northeast Ethiopia, Oromia Special Zone, Amhara National Regional State, 331 km away from north of Addis Ababa. The hospital is the only general hospital in the zone and currently serving about 1.2 million catchment population from all population in the zone and nearby districts. An institution based retrospective cross sectional study was conducted from March 1 to April 20, 2019 by reviewing a 1 year medical records (from January 1, 2018 to December 31, 2018) of pregnant women attending MCH clinic of the hospital.

#### Study population

All medical records of pregnant women who attended MCH clinic of KGH from Jan 1, 2018 to Dec 31, 2018 were taken as a study population.

#### Sample size determination and sampling technique

By using single population proportion formula, the sample size was determined as follows:

$${\text{n}} = \frac{{{\text{z}}^{ 2} {\text{p }}\left( { 1- {\text{p}}} \right)}}{{{\text{d}}^{2} }} = \frac{{\left( { 1. 9 6} \right)^{ 2} *\left( {0. 8 7 5} \right) \, \left( { 1- \, 0. 8 7 5} \right)}}{{\left( {0.0 3} \right)^{ 2} }} = 467,$$

where n is the desired sample size for population > 10,000, Z is the confidence level at 95% (1.96), P is the proportion of drug use during pregnancy, 87.5% [9], d is the degree of accuracy desired (marginal error) is 0.03. Since the number of pregnant women who attended MCH of KGH during the review period was 600 (< 10,000), correction formula was used to determine final sample size

$${\text{nf}} = \frac{\text{ni}}{{1 + \frac{{\text{ni}}}{{\text{N}}}}} = 467/(1 + 467/600) = 263$$

where nf is the final sample size, ni is the initial sample size, and N is the study population. Systematic random sampling was employed to select the study units.

#### Data collection processes and data quality control

The data abstraction format contained information regarding on obstetric history (age, trimesters of pregnancy, gravidity, number of visits), clinical and drug related data (common maternal disorders, common classes of drugs in each trimesters, and prescribed medications) and FDA pregnancy risk category. Pretest was done at Bati primary hospital located in the nearby town.

#### Data processing and analysis

The data were coded, entered and analyzed by using Statistical Package for Social Sciences (SPSS) version 23 (IBM statistics, Armonk, NY, USA).

### Result

#### Obstetric history of pregnant women

A total of 263 pregnant women medical records were reviewed for this study. The average maternal age in the study was 26.5 years. The majority of women expected to have child had 25–34 years of age. Most pregnant women 166 (63.2%) were in the third trimester and more than half (51.3%) were multigravida and started their ANC visits after the 24th week of their pregnancy (55.1%). Out of 263 pregnant women, 145 (55.1%) had four to six antenatal visits (Table 1).

**Table 1:** Obstetric history of pregnant women attending MCH clinic of KGH, Northeast Ethiopia from January 1, 2018 to December 31, 2018

Variables	Frequency	Percentage
Age (in years)		
15–24	90	34.2
25–34	162	61.6
35–50	11	4.2
Trimesters of pregnancy		
1st trimester	38	14.5
2nd trimester	59	22.4
3rd trimester	166	63.1
Gravidity		
Primigravida	38	14.5
Secundum gravida	90	34.2
Multigravida	135	51.3
Number of MCH visits		
< 3	103	39.2
4–6	145	55.1
> 7	15	5.7

#### Common maternal disorders

Anemia and vitamin deficiency is the most common maternal disorder 229 (87.07%) followed by intestinal parasite 14 (5.32%), nausea and vomiting 12 (4.56%), dyspepsia 11 (4.23%), and urinary tract infections 8 (3.04%) (Table 2).

**Table 2:** Common maternal disorders of pregnant women attending MCH clinic of KGH, Northeast Ethiopia from January 1, 2018 to December 31, 2018

Maternal disorders	Frequency	Percentage
Anemia and vitamin deficiency	229	87.07
Vaginal bleeding	2	0.76
AFI	6	2.28
Pneumonia	1	0.38
Malaria	1	0.38
Cough and cold	4	1.52
Dyspepsia	11	4.23
Gastritis	1	0.38
UTI	8	3.04
Nausea and vomiting	12	4.56
Intestinal parasite	14	5.32
HIV/AIDS	4	1.52
Diabetes mellitus	3	1.14
Amebiasis and gardiasis	2	0.76
Others	7	2.66
Total	305	116%

*AFI* acute febrile illness, *UTI* urinary tract infections, *HIV/AIDS* human immune virus/acquired immunodeficiency syndrome

#### Common classes of drugs and prescribed medications in each Trimester

From a total of 263 pregnant women, 234 had taken at least one drug with 88.97% prevalence of drug utilization, for which a total of 430 drugs were prescribed. Among these, supplemental drugs 297 (69.1%) were the most frequently prescribed drugs followed by anti-bacterial 35 (8.1%) and analgesics 24 (5.6%). The maximum number of drugs were prescribed in the second trimester 162 (37.67%) followed by third trimester 143 (33.26%) and first trimester 125 (29.07%). From antibiotics, amoxicillin, ceftriaxone and amoxicillin/clavunilic acid were mostly prescribed whereas paracetamol and diclofenac were frequently prescribed analgesics (Table 3).

**Table 3:** Medications used by pregnant women at different trimesters and their US FDA pregnancy risk category in KGH, Northeast Ethiopia from January 1, 2018–December 31, 2018

Class of drugs	Drug name	1st trimester frequency	2nd trimester frequency	3rd trimester frequency	Total frequency	FDA risk category
Antibiotics	Amoxicillin	5	3	2	10	B
	Ceftriaxone	2	5	2	9	B
	Cephalexin	1	2	2	5	B
	Amoxicillin/clavulinic	3	1	2	6	B
	Ampicillin	3	2	0	5	B
Supplements	Iron	20	80	78	178	A
	Folic acid	67	32	20	119	A
Analgesics	Paracetamol	9	5	3	17	B
	Diclofenac	0	0	5	5	D
	Ibuprofen	0	2	0	2	D
	Tramadol	0	1	0	1	C
GI drugs	Plasil	11	2	1	14	B
	Omeprazole	0	10	0	10	C
	Cimetidine	0	2	0	2	B
Other antimicrobials	Coartem	0	0	1	1	C
	Clotrimazole	3	1	4	8	B
	Mebendazole	0	2	12	14	C
	Metronidazole	0	4	4	8	C
DM drugs	Insulin	1	2	0	3	B
ART drugs	AZT/3TC/NVP	0	1	2	3	B
Iv fluids	N/S, R/L	0	5	5	10	C
	Total	125	162	143	430	

#### Summary of FDA drug risk category

Out of 430 drugs prescribed for pregnant women, 297 (69.1%) drugs were from FDA drug risk category A; 82 (19.1%) drugs from category B; 54 (12.6%) drugs from category C; and the rest 7 (1.6%) drugs from category D. There was no any drug from category X (Additional file 1: Table S1).

The average number of drugs per pregnant women who had at least one drug was found to be 1.84. Prevalence of drug utilization among pregnant women in this study was 88.97%. On the other hand, percentage of encounters with antibiotics prescribed was 35 (8.14%) whereas percentage of encounters with injections was 37 (8.6%). Moreover, the percentage of drugs prescribed with generic name was 412 (95.8%) (Additional file 2: Table S2).

### Discussion

Out of 263 pregnant women who attended MCH clinic of a hospital, the mean age was 26.5 (± 6.2) years and the majority were between 25 and 34 years which represents the normal reproductive age groups. This finding is line with a report by Legesse et al. [7]. More than half of pregnant women had four to six antenatal visits and most of them were in third trimester and started their antenatal care visits after the 24th week of their pregnancy. This finding is not in accordance with the reports by Fanta et al. [10] and Chanie et al. [8]. However, considerable numbers of pregnant women were in the first and second trimester where the critical periods of organogenesis and organ development occur and drug-induced teratogenicity is assumed to reach highest.

The prevalence of drug utilization in this study was found to be 88.97%, comparable with prevalence of 85.1% in eastern Ethiopia, Harar [11], 87.5% in northern Ethiopia [9] and 88.4% in north west Ethiopia, Bahirdar [12]. But this value is lower than a study in Nekemte (96%) [13].

Most common diseases recorded were anemia and vitamin deficiency, intestinal parasite, nausea and vomiting, dyspepsia, urinary tract infections and acute febrile illness. Hence, the majority of medications prescribed were drugs used against these problems such as iron salts and folic acid, antibiotics such as amoxicillin, ceftriaxone, metronidazole and amoxicillin/clavunilic acid, gastrointestinal drugs like metoclopramide, mebendazole and omeprazole. All of these drugs were either from FDA category A, B or C. The result of study was relatively higher than study reported in Nigeria [14]. This may be because of difference in maternal health problems such that intestinal parasite was among commonly presented maternal disorders in this study that increase percentage of drug utilization.

The average number of drugs per pregnant women in this study was 1.84 which indicated that it was slightly out of range of standard set by WHO (1.6–1.8) [15] as compared to the reports from studies conducted at Ayder referral hospital, Mekele and Hiwot Fana specialized university hospital, Harar (2.17, 1.21) respectively [3, 11].

Looking at the medications prescribed for pregnant women, supplemental drugs utilization accounted 69% of the total drugs, which is higher than a study conducted in eight rural districts of Ethiopia where only 35.4% of pregnant women used iron supplements [16] and lower than study conducted at Addis Ababa [17] and Harar city [11] which showed that more than 90% and 84.88% of the mothers were supplemented with at least one iron/folic acid supplement respectively. Much has to be done to create awareness, access and improve supplemental drugs utilization to almost every pregnant mother.

The majority of drugs were from category A (69.1%). This finding was far higher than a study conducted at Adama referral hospital [18] and Fiche primary hospital, Ethiopia [10] and a study done in Sao Paulo [19] which indicated that 13.6%, 20.83% and 20.55% drugs were from category A respectively. However, this finding was in line with a study done in Swaziland that reported 64.9% drugs were under category A. Whereas the current study was lower than studies conducted in Hiwot Fana Specialized University Hospital (84.88%) [11], in different health facilities of Southern Tigray region (87.7%) [9] and India (91.13%) [20]. The present study also showed that 19.1% of drugs were from category B, which was lower than a study conducted in Jimma [18] and Adama [21] where 60.2% and 62.4% were prescribed from category B.

Some group of drugs from category C (10.2%) and D (1.6%) were prescribed in this study. These values were lower than a study conducted at Addis Ababa where 21.43% and 16.96% drugs were from category C and D respectively [22]. There were not drugs from category C and D prescribed during first trimester, but considerable number of drugs was prescribed during the second and third trimesters. According to present study, diclofenac and ibuprofen (category D drugs) were used in third trimester for pregnant women to manage pain; however, this can cause early closure and constriction of ductus arteriosus with subsequent neonatal pulmonary hypertension and transient right-sided hypertrophic cardiomyopathy [23]. Other drugs such as mebendazole, metronidazole, tramadol, and artemether/lumefantrine are only recommended for use during pregnancy when benefit outweighs risk.

Besides, the present study showed that the percentage of drugs prescribed by the generic name was found to be 95.8%, which was comparable to a study done in Fiche Hospital [10] where 94.08% of the prescriptions were generic name drugs. Percentage of encounters with injections prescribed was 8.6%, less than a standard set by WHO [15]. 8.14% of the total prescribed medications were antibiotics. Among these, amoxicillin, ceftriaxone and amoxicillin/clavunilic acid were commonly prescribed which belongs to FDA category B drugs and are safe.

### Conclusion

Supplemental drugs (those from category A) constituted the majority of drugs utilized by pregnant women. Besides, approximately one third of the pregnant women were prescribed from category B, C and D drugs. In this study, diclofenac and ibuprofen (FDA category D drugs) were used for pain management in late pregnancy, but these drugs are known to cause premature closure of ductus arteriosus and subsequent neonatal pulmonary hypertension. Hence, prescription of drugs during pregnancy should always take into account FDA pregnancy risk category, trimester or the gestational period, and the risk–benefit ratio of drugs.

## Limitation of the study

- There was insufficient drug information on patients’ medical records and some patient medical records didn’t have diagnosis for the prescribed drugs at all.
- Retrospective nature of the study may not address the temporal r/ship b/n the drug exposure and the pregnancy risk.
- The retrospective study did not examine over-the-counter medications and herbal medicines that the pregnant women may self medicate but still might produce teratogenicity.
- Besides, the study utilized the former FDA classification system which seems overly simplified despite the fact that the new narrative system has no cut-off point and couldn’t be studied with retrospective chart review.

## Supplementary information

**Additional file 1: Table S1.** Frequency distribution of FDA drug category of the drug prescribed at different trimesters in KGH, Northeast Ethiopia from January 1, 2018- December 31, 2018.

**Additional file 2: Table S2.** Frequency and percentage distribution of prescribing pattern indicators in each trimester in KGH, Northeast Ethiopia in reference to WHO standards.

**Additional file 3: Table S3.** Meaning of United States-Food and Drug Administration pregnancy drugs risk categorization (A–X letters system).

## Data Availability

All the data used for the study is contained within the manuscript and Additional Additional file 1: Table S1, Additional file 2: Table S2 and Additional file 3: Table S3.

## Abbreviations

AFI: acute febrile illness
AIDS: acquired immune deficiency syndrome
ART: anti retroviral therapy
KGH: Kemisse General Hospital
HIV: human immune virus
MCH: Maternal and Child Health
US-FDA: United State of Food & Drug Administration
WHO: World Health Organization
UTI: urinary tract infection
