# Supplementary material for: Prescription drug use and potential teratogenicity risk among pregnant women attending maternal and child health clinic of Kemisse General Hospital, Northeast, Ethiopia
Source: BMC Res Notes. 2019 Sep 18;12:592. doi: 10.1186/s13104-019-4641-1 (PMC6751805; doi:10.1186/s13104-019-4641-1)
Supplement: Supplementary file 1 — Additional file 1: Table S1. Frequency distribution of FDA drug category of the drug prescribed at different trimesters in KGH, Northeast Ethiopia from January 1, 2018- December 31, 2018. [file 13104_2019_4641_MOESM1_ESM.docx]

**Table S1: Frequency distribution of FDA drug category of the drug prescribed at different trimesters in KGH, Northeast Ethiopia from January 1, 2018- December 31, 2018.**

| US FDA risk category | 1^st^ trimester | 2^nd^ trimester | 3^rd^ trimester | Total |
| --- | --- | --- | --- | --- |
| A | 87 | 112 | 98 | 297 (69.1%) |
| B | 38 | 26 | 18 | 82 (19.1%) |
| C | 0 | 22 | 22 | 54 (10.2%) |
| D | 0 | 2 | 5 | 7 (1.6%) |
| Total | 125 | 162 | 143 | 430 |
