# Supplementary material for: Prescription drug use and potential teratogenicity risk among pregnant women attending maternal and child health clinic of Kemisse General Hospital, Northeast, Ethiopia
Source: BMC Res Notes. 2019 Sep 18;12:592. doi: 10.1186/s13104-019-4641-1 (PMC6751805; doi:10.1186/s13104-019-4641-1)
Supplement: Supplementary file 2 — Additional file 2: Table S2. Frequency and percentage distribution of prescribing pattern indicators in each trimester in KGH, Northeast Ethiopia in reference to WHO standards. [file 13104_2019_4641_MOESM2_ESM.docx]

**Table S2: Frequency and percentage distribution of prescribing pattern indicators in each trimester in KGH, Northeast Ethiopia in reference to WHO standards.**

| Prescribing indicators | 1^st^ trimester | 2^nd^ trimester | 3^rd^ trimester | Total | WHO standard |
| --- | --- | --- | --- | --- | --- |
| Percentage of drugs prescribed with generic name | 114 | 159 | 139 | 412(95.8%) | 100% |
| Percentage of encounters with antibiotics prescribed | 14 | 13 | 8 | 35 (8.14%) | < 30 (20–26.8%) |
| Percentage of encounters with injections prescribed | 11 | 15 | 11 | 37(8.6%) | (13.4–21.1%) |
| Average No of drugs per prescription = 1.84 (430 drugs / 234 encounters) | | | | | ≤ 2 (1.6–1.8) |
