# Supplementary material for: Prescription drug use and potential teratogenicity risk among pregnant women attending maternal and child health clinic of Kemisse General Hospital, Northeast, Ethiopia
Source: BMC Res Notes. 2019 Sep 18;12:592. doi: 10.1186/s13104-019-4641-1 (PMC6751805; doi:10.1186/s13104-019-4641-1)
Supplement: Supplementary file 3 — Additional file 3: Table S3. Meaning of United States-Food and Drug Administration pregnancy drugs risk categorization (A–X letters system). [file 13104_2019_4641_MOESM3_ESM.docx]

| FDA pregnancy categories | Their meaning |
| --- | --- |
| Category A | Adequate and well-controlled studies have failed to demonstrate a risk to the fetus in the first trimester of pregnancy (and there is no evidence of risk in later trimesters). |
| Category B | Animal reproduction studies have failed to demonstrate a risk to the fetus and there are no adequate and well-controlled studies in pregnant women. |
| Category C | Animal reproduction studies have shown an adverse effect on the fetus and there are no adequate and well-controlled studies in humans, but potential benefits may warrant use of the drug in pregnant women despite potential risks. |
| Category D | There is positive evidence of human fetal risk based on adverse reaction data from investigational or marketing experience or studies in humans, but potential benefits may warrant use of the drug in pregnant women despite potential risks. |
| Category X | Studies in animals or humans have demonstrated fetal abnormalities and/or there is positive evidence of human fetal risk based on adverse reaction data from investigational or marketing experience, and the risks involved in use of the drug in pregnant women clearly outweigh potential benefits. |

**Table S3: Meaning of United States-Food and Drug Administration pregnancy risk categorization (old A-Z letters system).**

Source: [Content and Format of Labeling for Human Prescription Drug and Biological Products; Requirements for Pregnancy and Lactation Labeling](http://frwebgate.access.gpo.gov/cgi-bin/getdoc.cgi?dbname=2008_register&docid=fr29my08-33.pdf) (Federal Register/Vol. 73, No. 104/Thursday, May 29, 2008)
